# Phenome-wide investigation of the causal associations between childhood BMI and adult trait outcomes: a two-sample Mendelian randomization study

**DOI:** 10.1186/s13073-021-00865-3

**Published:** 2021-03-26

**Authors:** Shan-Shan Dong, Kun Zhang, Yan Guo, Jing-Miao Ding, Yu Rong, Jun-Cheng Feng, Shi Yao, Ruo-Han Hao, Feng Jiang, Jia-Bin Chen, Hao Wu, Xiao-Feng Chen, Tie-Lin Yang

**Affiliations:** 1 Key Laboratory of Biomedical Information Engineering of Ministry of Education, Biomedical Informatics & Genomics Center, School of Life Science and Technology, Xi’an Jiaotong University, Xi’an, 710049 China; 2 National and Local Joint Engineering Research Center of Biodiagnosis and Biotherapy, The Second Affiliated Hospital, Xi’an Jiaotong University, Xi’an, 710004 China

**Keywords:** Childhood BMI, Mendelian randomization, Adult outcome, Causal

## Abstract

**Background:**

Childhood obesity is reported to be associated with the risk of many diseases in adulthood. However, observational studies cannot fully account for confounding factors. We aimed to systematically assess the causal associations between childhood body mass index (BMI) and various adult traits/diseases using two-sample Mendelian randomization (MR).

**Methods:**

After data filtering, 263 adult traits genetically correlated with childhood BMI (*P* < 0.05) were subjected to MR analyses. Inverse-variance weighted, MR-Egger, weighted median, and weighted mode methods were used to estimate the causal effects. Multivariable MR analysis was performed to test whether the effects of childhood BMI on adult traits are independent from adult BMI.

**Results:**

We identified potential causal effects of childhood obesity on 60 adult traits (27 disease-related traits, 27 lifestyle factors, and 6 other traits). Higher childhood BMI was associated with a reduced overall health rating (*β* = − 0.10, 95% CI − 0.13 to − 0.07, *P* = 6.26 × 10^−11^). Specifically, higher childhood BMI was associated with increased odds of coronary artery disease (OR = 1.09, 95% CI 1.06 to 1.11, *P* = 4.28 × 10^−11^), essential hypertension (OR = 1.12, 95% CI 1.08 to 1.16, *P* = 1.27 × 10^−11^), type 2 diabetes (OR = 1.36, 95% CI 1.30 to 1.43, *P* = 1.57 × 10^−34^), and arthrosis (OR = 1.09, 95% CI 1.06 to 1.12, *P* = 8.80 × 10^−9^). However, after accounting for adult BMI, the detrimental effects of childhood BMI on disease-related traits were no longer present (*P* > 0.05). For dietary habits, different from conventional understanding, we found that higher childhood BMI was associated with low calorie density food intake. However, this association might be specific to the UK Biobank population.

**Conclusions:**

In summary, we provided a phenome-wide view of the effects of childhood BMI on adult traits. Multivariable MR analysis suggested that the associations between childhood BMI and increased risks of diseases in adulthood are likely attributed to individuals remaining obese in later life. Therefore, ensuring that childhood obesity does not persist into later life might be useful for reducing the detrimental effects of childhood obesity on adult diseases.

**Supplementary Information:**

The online version contains supplementary material available at 10.1186/s13073-021-00865-3.

## Background

Obesity is a worldwide health problem. The prevalence of adult obesity has increased dramatically since the 1980s [1]. It is particularly worrisome that the rate of increase in childhood obesity has been nearly double that in adults [1]. Childhood overweight and obesity often persist in adulthood, which increases the risks of premature mortality and physical morbidity across the lifespan [2].

Compelling observational studies have reported that childhood obesity is associated with the risk of many complex diseases in adulthood, such as coronary artery disease (CAD) [3], cancers [4], diabetes [5], and polycystic ovary syndrome symptoms [6]. However, results from observational studies are unable to fully account for confounding factors (e.g., socioeconomic status). Therefore, whether the relationship is causal is uncertain.

**Fig. 1:**
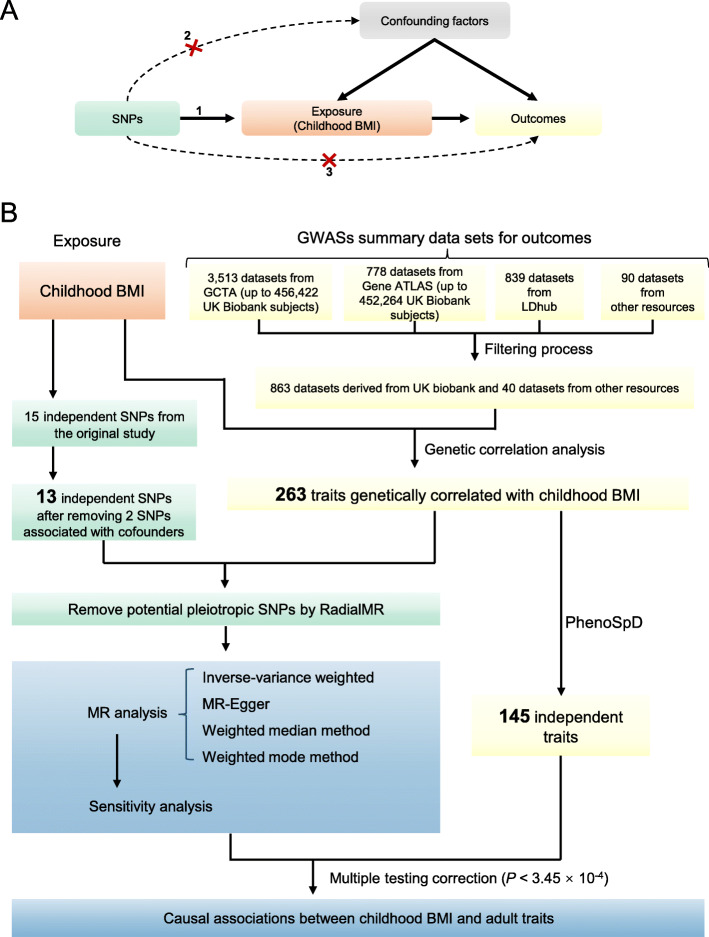
**a** Schematic diagram of an MR analysis. Since genetic alleles are independently segregated and randomly assigned, SNPs are not associated with confounding factors that may bias estimates from observational studies. Three assumptions of MR are as follows: (1) the selected instrument is predictive of the exposure, (2) the instrument is independent of confounding factors, and (3) there is no horizontal pleiotropy (the instrument is associated with the outcome only through the exposure). **b** The analysis pipeline of the current study

Mendelian randomization (MR), which uses genetic markers of the exposure as instruments, is now widely used to assess the causal relationship between exposure and outcome [7]. As shown in Fig. 1a, MR must satisfy three assumptions [7]: (1) the selected instruments must be associated with the exposure, (2) the instruments must not be associated with confounding factors, and (3) the instruments must influence the outcome only through the exposure (no horizontal pleiotropy exists). Conventionally, one-sample MR could be performed by using the two-stage least squares analysis method. For example, a previous study [8] using one-sample MR showed that abdominal adiposity might have a causal unfavorable effect on cardiometabolic risk factors in children and adolescents. Recently, two-sample MR analysis methods using summary-level GWAS data have been developed [9]. With a large amount of GWAS summary data deposited in public databases, two-sample MR analysis provides a cost-efficient way to investigate the potential causal effects of childhood obesity on adult traits. Using this method, previous studies have demonstrated the causal adverse effects of childhood body mass index (BMI) on adult cardiometabolic diseases [10] and osteoarthritis [11]. Using SNPs associated with adult BMI as instruments, two recent MR phenome-wide association studies [12, 13] have shown the causal effects of adult obesity on many other traits/diseases. The causal effects of childhood obesity are suspected but have not been systematically characterized.

Another interesting question is whether the causal effect of childhood BMI on the later health outcomes is independent from adult BMI. It was reported that childhood obesity was associated with an increased risk of multiple comorbidities in adulthood even if the obesity did not persist [14]. However, a recent study [15] showed that the observational association between childhood overweight and adult type 2 diabetes (T2D) only hold if the overweight continued until puberty or later ages. Multivariable MR [16] can be used to determine whether several exposures affect an outcome through the same pathway or whether the exposures have independent effects. A study [17] using multivariable MR showed that the causal adverse effects of large body size in early life on CAD and T2D is depend on adult body size. Systematically assessing the influences of childhood BMI on adult traits and whether these effects are independent from adult BMI might be useful for subsequent decision on the timing of preventive strategies.

In this study, we performed a MR phenome-wide association study to assess the causal effects of childhood BMI on adult traits/diseases using 2-sample MR with current available GWAS summary data (data collected before August 2019). Multivariable MR was also used to determine the independent effects of childhood BMI after accounting for adult BMI. Our results offer a systemic view of the causal effects of childhood BMI on adult traits.

## Methods

The outline of the experimental approach used in this study is shown in Fig. 1b. The STROBE-MR checklist (https://peerj.com/preprints/27857/) [18] was used for reporting this work.

### Summary data resources

#### Childhood BMI

The childhood BMI GWAS summary dataset was from the Early Growth Genetics consortium (http://egg-consortium.org/childhood-bmi.html, “EGG_BMI_HapMap_DISCOVERY.txt.gz”). The phenotype used in this GWAS was sex- and age-adjusted standard deviation scores of childhood BMI at the latest time point (oldest age) between 2 and 10 years [19]. The GWAS included 47,541 European children in total.

#### Adulthood outcomes

GWAS summary data were obtained from the following resources: (1) 3513 GWAS summary data on up to 456,422 array-genotyped and imputed UK Biobank individuals (aged between 40 and 69 at recruitment) from the Genome-wide Complex Trait Analysis (GCTA) website; (2) 778 GWAS summary datasets for up to 452,264 UK Biobank individuals from the Gene ATLAS database (http://geneatlas.roslin.ed.ac.uk/); and (3) 839 GWAS datasets from the LDhub GWAShare Center (http://ldsc.broadinstitute.org/); 4) 90 datasets from various other resources (Additional file 1: Table S1). All datasets were collected before May 2019.

Next, we filtered the GWAS summary datasets first using the following criteria:

1. GWAS with small sample size and limited statistical power might fail to detect SNP-trait associations [20]. To avoid potential horizontal pleiotropy, it is necessary to make sure that the outcome data we collected have enough sample size to detect SNP-trait associations. Here we only kept data sets with *N* > 50,000 and both cases and controls are > 10,000 for binary phenotypes. When the significance threshold is *P* < 5 × 10^−8^, GWAS with this sample size has over 90% power to detect SNPs with explained phenotypic variance portion of over 1 × 10^−3^. The statistical power was calculated using the formula presented in the work of Visscher et al. [20]. The same cutoff has also been used in a previous study which aimed to analyze pleiotropy in multiple traits [21].
2. Confounding by ancestry could occur if instruments associated with exposure had different frequencies in different ethnic groups [22]. The exposure data we used for childhood BMI is from the European ancestry. Therefore, we only kept the GWAS summary data set which is based on European population or > 80% of the samples are European.
3. Exclude sex-specific GWAS, unless the trait is only available for a specific sex (e.g., breast cancer).
4. Exclude adolescent traits, parent or sibling traits (e.g., illnesses of father). We also removed traits related to adult obesity, since 11 of the 15 childhood BMI SNPs are in linkage disequilibrium (LD) with adult BMI variants.
5. If a trait has more than one GWAS dataset, we only kept the dataset with the greatest number of subjects for this trait.

Finally, a total of 903 datasets remained, including 863 datasets specifically for the UK Biobank population (Additional file 2: Fig. S1). For all 903 datasets, the URLs for detailed phenotype description and data access are listed in Additional file 1: Table S2. All outcomes were recoded to make sure the variables followed increasing patterns. For example, overall health rating was originally coded from 1 to 4 to refer excellent, good, fair, and poor, respectively. Under such situation, an allele positively associated with this trait is actually a risk factor of overall health rating. To avoid misunderstanding, we recoded these traits by changing the plus or minus sign of the beta value in the association results.

### Estimated standardized effect size of SNPs

To enable comparison of effect sizes across studies, we obtained the estimated standardized effect size (*β*) and standard error (se) as a function of minor allele frequency and sample size as described previously [23] using the following equation:

$$ \beta =\frac{z}{\sqrt{\ 2p\left(1-p\right)\left(\mathrm{n}+{z}^2\ \right)}}, se=\frac{1}{\sqrt{\ 2p\left(1-p\right)\left(\mathrm{n}+{z}^2\ \right)}} $$

where *z* can be calculated as *β*/se from the original summary data, *p* is the minor allele frequency, and *n* is the total sample size.

### Genetic correlation analyses

As a phenome-wide study, our hypothesis-free MR analyses with many independent statistical tests might suffer from the problem of multiple testing burden [24]. On the other hand, if two traits are causally related and both of them have non-zero heritability, there should be genetic correlations between them [24]. Therefore, to solve the problem of multiple testing burden, we firstly screened the large publicly available GWAS summary results for evidence of genetic correlation with childhood BMI using LD score regression [25]. Formal MR analyses were subsequently performed to assess the causal effects. As an analytical strategy to mine the phenome [24], this analysis process has also been used in a previous study [26]. We used genetic correlation analysis to select data potentially associated with childhood BMI for further MR analysis; therefore, we used *P* < 0.05 as the cutoff to preserve all datasets with suggestive evidence. All traits were classified into three main categories—lifestyle factors, disease-related traits, and others. All disease-related traits were further classified according to the International Classification of Diseases 11th Revision (ICD-11) [27].

### Instruments selection

Fifteen independent SNPs with *P* < 5 × 10^−8^ identified from the original GWAS study [19] for childhood BMI were used as instruments (Additional file 1: Table S3). The genetic risk score of these SNPs explained 2.0% of the variance in childhood BMI [19]. To avoid potential confounding, we looked up each instrument SNP and their proxies (r^2^ > 0.8) in the PhenoScanner GWAS database (http://phenoscanner.medschl.cam.ac.uk) [28, 29] to assess any previous associations (*P* < 0.0033 (0.05/15)) with 4 plausible confounders selected based on previously published studies: birth weight [19, 30, 31], years of educational attainment and age completed full time education [32–34], and maternal smoking around birth [35–38]. Two SNPs were associated with a potential confounder (rs12041852, maternal smoking around birth, *P* = 7.43 × 10^−5^; rs12507026 (in LD with rs13130484), years of educational attainment, *P* = 0.0028), resulting a set of 13 SNPs for further analysis. In addition to the GWAS which reported these SNPs, 11 of the 13 loci have also been reported to be associated with childhood obesity in other previously published studies (Additional file 1: Table S4). For each outcome, we also used the RadialMR [39] package to further exclude outlying pleiotropic SNPs. RadialMR [39] identified outlying genetic instruments via modified Q-statistics. Among the 13 SNPs, 9 SNPs were in LD with adult BMI variants (Additional file 1: Table S3). The effect sizes (se) of the rest 4 SNPs were 0.042 (0.007), 0.045 (0.008), 0.041 (0.007), and 0.139 (0.025) respectively.

### MR analyses

We used four complementary methods of two-sample MR (inverse variance weighted (IVW) method, MR-Egger method, weighted median method, and weighted mode method) to estimate the causal effects. They make different assumptions about horizontal pleiotropy. When the horizontal pleiotropy is balanced (i.e., the pleiotropic effects are independent of SNP-exposure effects), there should be no bias in the effect derived from MR. If the horizontal pleiotropic effects are biasing the estimate in the same direction (directional pleiotropy), the causal estimates will be biased (except for the MR-Egger method).

The IVW method assumes balanced pleiotropy [40]. We obtained the IVW estimate by meta-analyzing the SNP specific Wald estimates using multiplicative random effects. Cochran’s *Q* statistic [41] was used to check for the presence of heterogeneity, which can indicate pleiotropy. Cochran’s *Q* statistic [41] follows a *χ*^2^ distribution with *L* − 1 degrees of freedom (*L* refers to the number of instruments) under the null hypothesis of homogeneity.

The MR-Egger method is based on the INSIDE assumption (instrument strength independent of the direct effects) [40]. It requires that the SNPs’ potential pleiotropic effects are independent of the SNPs’ association with the exposure [40]. MR-Egger is also based on the no measurement error in the SNP exposure effects (NOME) assumption, which can be evaluated by the regression dilution I^2^ (GX) [42]. When *I*^2^ (GX) < 0.9, adjustment methods should be considered [42]. Therefore, simulation extrapolation (SIMEX) correction analysis was performed to estimate the causal effect when *I*^2^ (GX) < 0.9 [42]. The intercept term of the MR-Egger method represents an estimate of the directional pleiotropic effect [43]. We also calculated the Rucker’s *Q*′ statistic [44] to measure the heterogeneity in the MR-Egger analysis. Rucker’s *Q*′ follows a *χ*^2^ distribution with *L* − 2 degrees of freedom under the null hypothesis of no heterogeneity (*L* refers to the number of instruments) [44]. Generally, we have Rucker’s *Q*′ ≤ Cochran’s *Q* [44]. If the difference *Q* − *Q*′ is sufficiently extreme with respect to a *χ*^2^ distribution with the 1 degree of freedom, we would infer that directional pleiotropy is an important factor and MR-Egger model provides a better fit than the IVW method [45].

The weighted median method estimates the causal effect under the assumption that at least 50% of the total weight of the instrument comes from valid variants [46]. Compared with IVW and MR-Egger, this method has greater robustness to provide a consistent causal effect estimate even when up to 50% of the SNPs are invalid instruments [46]. The mode-based method provides a consistent effect estimate when the largest number of similar individual-instrument estimates come from valid instruments, even if the majority of instruments are invalid [47].

We also used MR pleiotropy residual sum and outlier (MR-PRESSO) global test [48] to detect horizontal pleiotropy. The analyses of the four MR methods were carried out using the TwoSampleMR package in R. We chose the main MR method as follows:

- If no directional pleiotropy was detected (*P* > 0.05 for tests of *Q*, MR-Egger intercept, *Q* − *Q*′ and MR-PRESSO), use IVW.
- If directional pleiotropy was detected and *P* > 0.05 for the test of *Q*′, use MR-Egger.
- If directional pleiotropy was detected and *P* < 0.05 for the test of Q′, use weighted median.

We also checked the consistency of the directions in all four MR methods. Only significant results with the same direction in all methods were remained to make sure the positive results we selected are robust under different assumptions.

Effect estimates are reported in *β* values for continuous outcomes and converted to ORs for dichotomous outcomes.

### Sensitivity analysis

For outcomes with significant MR analysis results, leave-one-out sensitivity analysis was carried out to check whether the causal association was driven by a single SNP. Over 95% of the outcome datasets we used are specifically for the UK Biobank population. To check whether the significant results could be replicated in other datasets, we performed MR analysis for 5 outcomes (CAD, disease count, hypertensive disease, osteoarthritis, and T2D) with available summary data from resources without UK Biobank participants. The datasets for disease count, hypertensive disease, and osteoarthritis were from the Genetic Epidemiology Research on Adult Health and Aging (GERA) cohort [49]. The datasets for CAD and T2D were obtained from the studies performed by Nikpay et al. [50] and Scott et al. [51], respectively. Detailed phenotype description and data access URLs are listed in Additional file 1: Table S5.

### Estimating the number of independent outcomes

As our analysis involved a large number of summary data, we expected that some of these outcomes might be highly correlated with each other. Therefore, we used PhenoSpD [52] to estimate the number of independent outcomes to correct for multiple testing. We used the LD score regression method [25] to create a correlation matrix between each outcome. The matrix was used as an input for PhenoSpD to assess the number of independent outcomes through matrix spectral decomposition. Suppose the number of independent outcomes is *n*, then the significant threshold was set as 0.05/*n* after multiple testing correction.

### Polygenic risk score (PRS) analyses for dietary habits with significant MR results

We used the SNP instruments in MR analysis as markers to construct PRSs for childhood BMI and adult BMI. UK Biobank samples were used as the target datasets. We only used samples with the ethnic background of European ancestry. Samples with missingness > 5% and mismatching phenotypic and genotypic sex and samples that have withdrawn consent were excluded. PRSs were calculated using the software PRsice [53]. The correlations between PRSs and dietary traits were tested with age, sex, and top 10 principal components as covariates. Logistic regression was used for binary phenotypes and linear regression was used for continuous phenotypes.

### Instruments for adult BMI and multivariable MR analysis

For outcomes with significant MR analysis results, we also carried out MR analyses for adult BMI. Over 95% of the outcome data we used were from the UK Biobank population. In two-sample MR analysis, overlap in participants between the exposure and outcome can cause bias towards the risk factor-outcome association [54]. Therefore, we used the adult BMI SNPs reported by Locke et al. [55] rather than the SNPs reported by Yengo et al. [56] since 65% (450,000/700,000) in Yengo et al. are the UK Biobank participants. To avoid potential confounding caused by ancestry [22], we only used the reported SNPs by Locke et al. from the European-descent individuals. Among the total 77 SNPs, one SNP (rs12016871) was not present in the 60 summary data sets of the outcomes, so we used the rest 76 independent SNPs as instruments (Additional file 1: Table S6). Multivariable MR analysis [16] was then used to determine whether childhood BMI and adult BMI affect the outcomes through the same pathway or whether they have independent effects. SNPs from the univariable MR analysis were used after performing linkage disequilibrium clumping to account for instrument correlation between the two sets.

### Reverse-direction MR analyses

For the 60 traits with significant causal effects, we also performed reverse-direction MR to assess potential reverse causal effects. For each exposure, we used the clumping algorithm in PLINK [57] to select independent SNPs for each trait (*r*^2^ threshold = 0.001, window size = 1 Mb and *P* < 5 × 10^−8^). The 1000G European data (phase 3) were used as the reference for LD estimation. For exposures with less than 3 significant SNPs available for MR, we used SNPs meeting a more relaxed threshold (*P* < 1 × 10^−5^). This relaxing statistical threshold method for genetic instruments has been used in previous MR studies [26]. The MR analyses process was the same as previously described.

## Results

### Genetic correlation analyses

According to the cross-trait LD score analyses, 263 outcomes showed genetic correlation with childhood BMI (Fig. 1b, Additional file 1: Table S2), including 249 outcomes specific for the UK Biobank population. We manually checked the cohorts involved in these outcomes and found that samples in these studies were not overlapped with those in the childhood BMI study. These outcomes (138 disease-related traits, 80 lifestyle factors, and 45 other traits) were subjected to subsequent MR analysis.

### Assessment of pleiotropy

The results of assessment of pleiotropy are shown in Additional file 1: Table S7. No significant evidence of pleiotropy was detected by the Cochran’s *Q* test and MR-PRESSO global test (*P* > 0.05). MR-Egger’s intercept test detected evidence of directional pleiotropy for 2 outcomes (*P* < 0.05, Additional file 1: Table S7, Additional file 2: Fig. S2A). The difference *Q* − *Q*′ is sufficiently extreme with respect to a *χ*^2^ distribution with the 1 degree of freedom in additional 7 outcomes (*P* < 0.05, Additional file 1: Table S7, Additional file 2: Fig. S2B). Since Rucker’s *Q*′ test did not detect evidence of heterogeneity in these 9 outcomes, MR-Egger was chosen as the main method for them. For the other outcomes without evidence of directional pleiotropy, we chose IVW as the main MR method.

The NOME assumption violation (*I*^2^ (GX) < 0.9) was detected in all outcomes (Additional file 1: Table S7). Therefore, we also carried out MR-Egger with SIMEX analyses.

### MR results

**Fig. 2:**
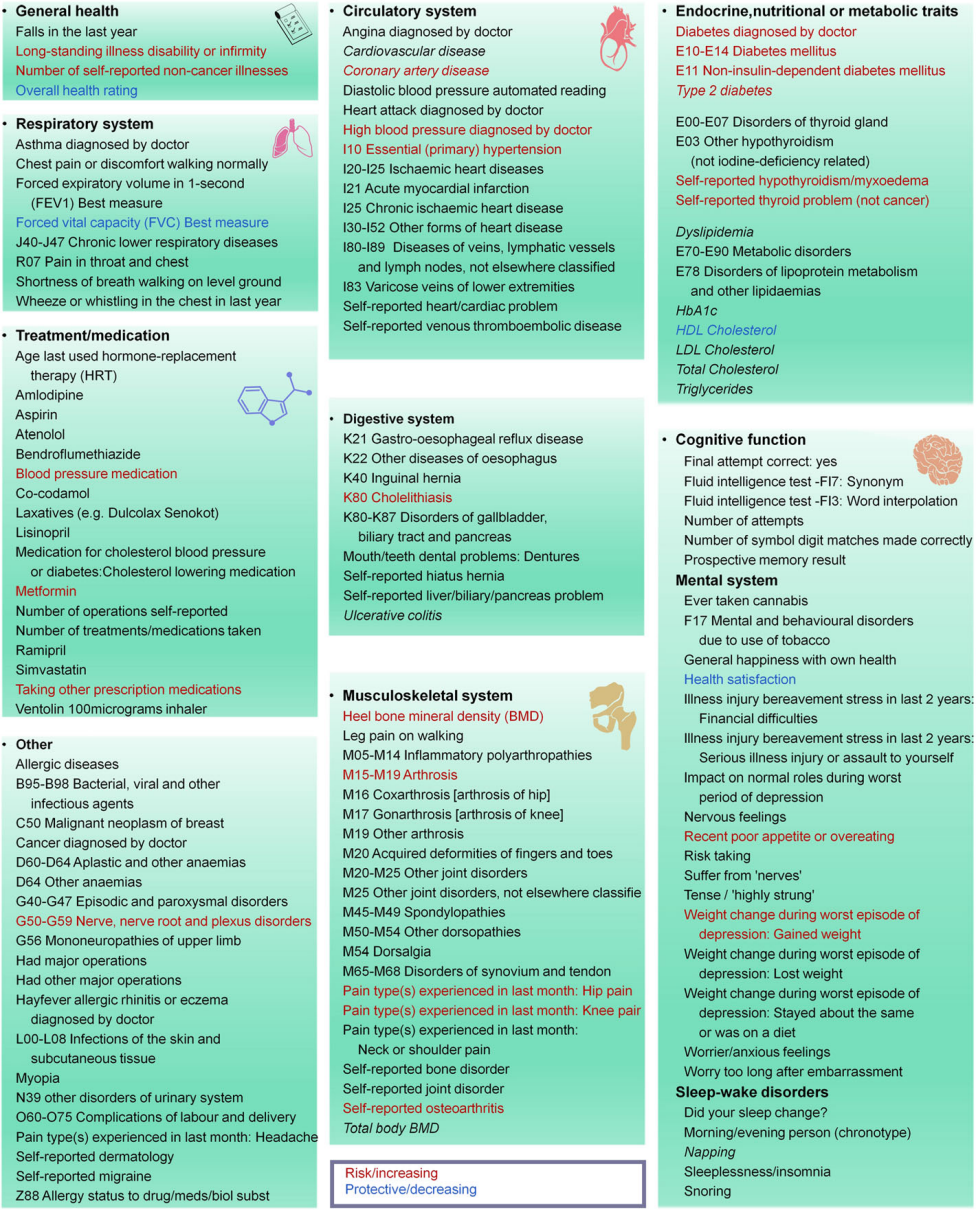
Summary view of the MR analysis results for the disease-related traits. Traits with significant positive associations with childhood BMI are shown in red. Traits with significant negative associations with childhood BMI are shown in blue. The other traits are shown in black. Traits from resources not specific to the UK Biobank population are shown in italic. For diseases from the UK Biobank population, those with pre-posed code (e.g., K80 Cholelithiasis) are obtained from clinical diagnoses. Diseases without pre-posed code were obtained from questionnaire. The URLs for detailed description for all phenotypes are listed in Additional file 1: Table S2

**Fig. 3:**
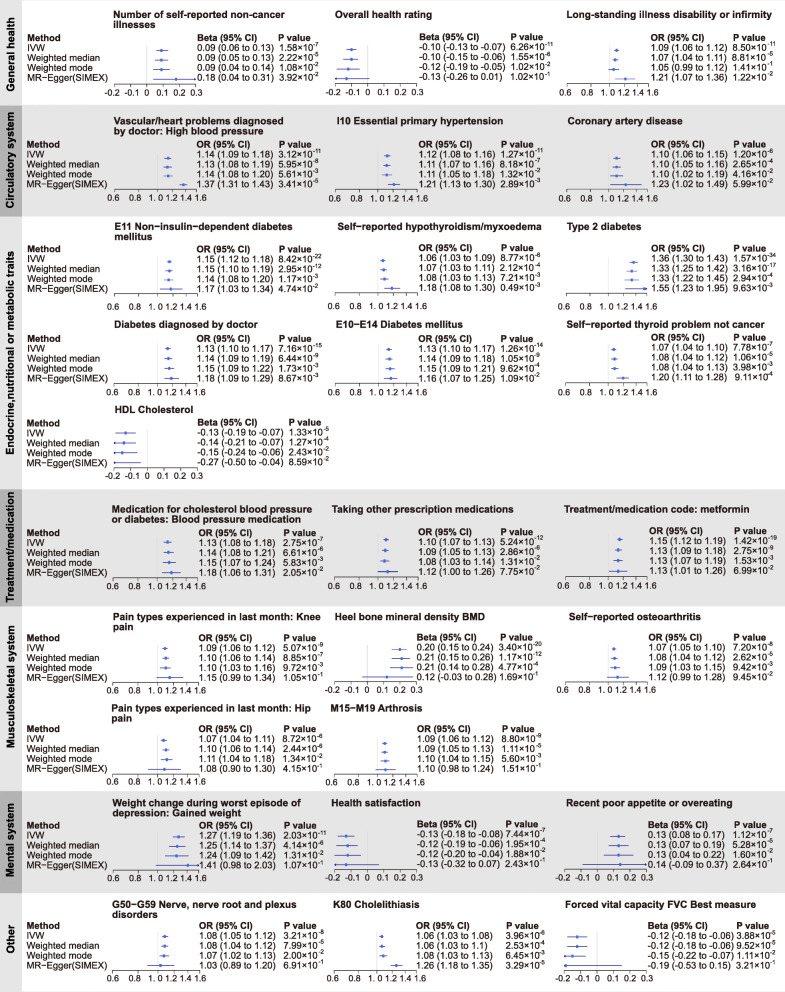
Summary Mendelian randomization (MR) estimates derived from the inverse-variance weighted, MR-Egger, weighted median, and weighted mode-based methods for the 27 disease-related traits. Childhood BMI was used as exposure and significant associations were detected for these traits

**Fig. 4:**
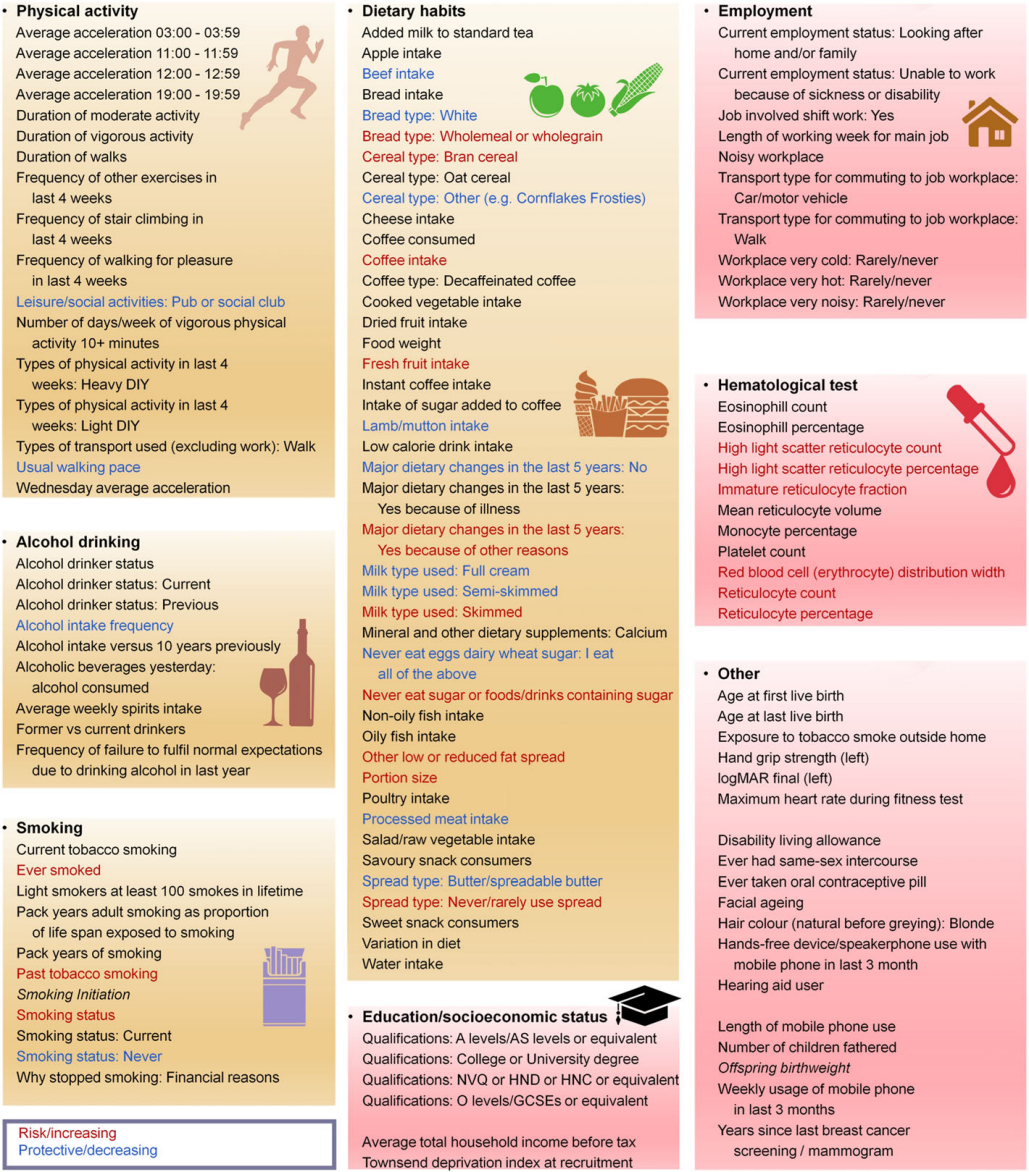
Summary view of the MR analysis results for the lifestyle factors and other traits. Traits with significant positive associations with childhood BMI are shown in red. Traits with significant negative associations with childhood BMI are shown in blue. The other traits are shown in black. Traits from resources not specific to the UK Biobank population are shown in italic. The URLs for detailed description for all phenotypes are listed in Additional file 1: Table S2

**Fig. 5:**
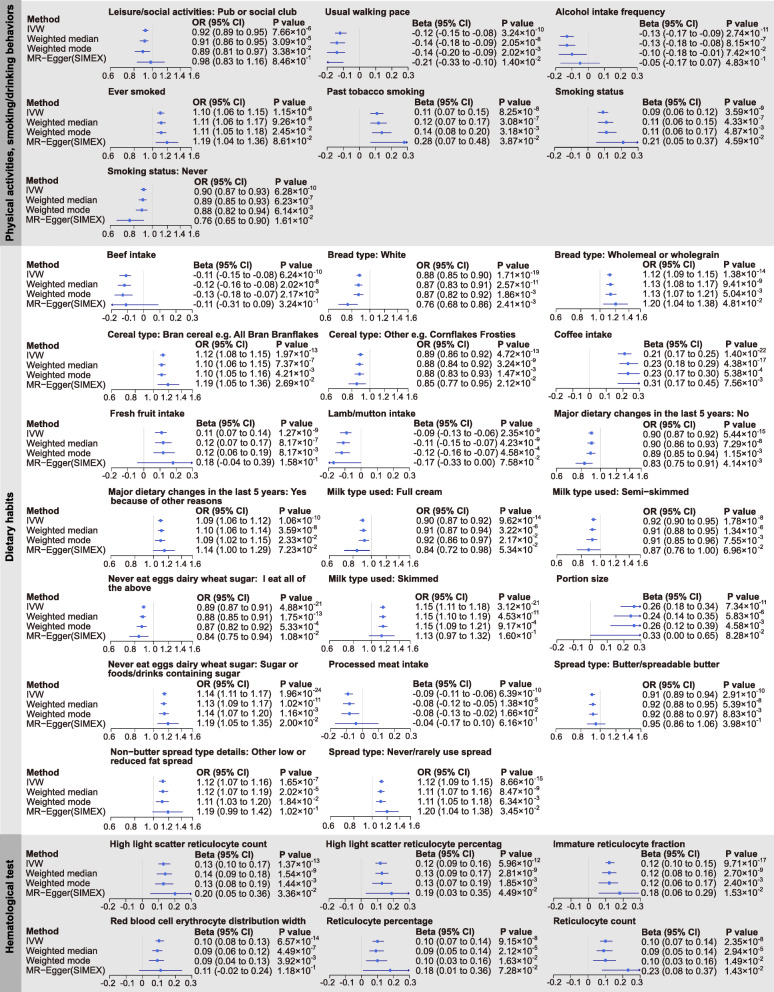
Summary Mendelian randomization (MR) estimates derived from the inverse-variance weighted, MR-Egger, weighted median, and weighted mode-based methods for the 27 lifestyle factors and 6 other hematological test traits. Childhood BMI was used as exposure and significant associations were detected for these traits

The results of PhenoSpD showed that the independent outcome number was 145, setting the Bonferroni *P* value threshold for our main MR analysis at *P* < 3.45 × 10^−4^ (0.05/145). In addition to multiple testing corrections of the main MR method, *P* < 3.45 × 10^−4^ of the weighted median method was also set as a cutoff to obtain confident results supported by at least two MR methods. Sixty significant associations were detected (Additional file 1: Table S8). A total of 27 disease-related traits, 27 lifestyle factors, and 6 other traits were included. For better illustration, we summarized the MR findings in Figs. 2, 3, 4, and 5.

The performances of the four methods were similar. Using the threshold of *P* < 3.45 × 10^−4^, the IVW and weighted median methods supported the causal associations between childhood BMI and all 60 traits. But the numbers of associations supported by the weighted mode and MR-Egger methods were only 1 and 3 outcomes, respectively. The difference may be due to the fact that the power of weighted mode and MR-Egger methods is smaller than that of the IVW and weighted median methods [47]. At the suggestive significant level of 0.05, 59 of the 60 associations were supported by at least three methods. The weighted mode and MR-Egger method detected the associations with 58 and 29 outcomes, respectively. This is consistent with the previous report that MR-Egger has the lowest power of the four methods to detect a causal effect [47].

### Childhood obesity is a risk factor for general health outcomes in adulthood

As shown in Figs. 2 and 3 and Additional file 1: Table S8, there is evidence that childhood BMI causally affects a total of 27 outcomes related to adult diseases, including 3 general health traits; 3 circulatory system traits; 7 endocrine, nutritional, metabolic traits; 5 musculoskeletal system traits; and 9 other traits.

#### Childhood BMI and general health

As shown in Fig. 3 and Additional file 2: Fig. S3, higher childhood BMI was associated with reduced overall health rating (*β* = − 0.10, 95% CI − 0.13 to − 0.07, *P* = 6.26 × 10^−11^) and an increased the number of self-reported non-cancer illnesses (*β* = 0.09, 95% CI 0.06 to 0.13, *P* = 1.58 × 10^−7^). One SD increase in childhood BMI was associated with 9% higher odds of long-standing illness disability or infirmity (OR = 1.09, 95% CI 1.06 to 1.12, *P* = 8.50 × 10^−11^). Leave-one-out analysis showed that no single SNP was driving the causal estimates (Additional file 2: Fig. S3). There was no association between childhood BMI and falls in last year (*P* > 0.05, Additional file 1: Table S8).

#### Childhood BMI and circulatory system traits

We found that a 1 SD increase in childhood BMI was associated with 9% higher odds of CAD (OR = 1.09, 95% CI 1.06 to 1.11, *P* = 4.28 × 10^−11^, Fig. 3, Additional file 2: Fig. S4). The other two circulatory system traits with significant associations are essential hypertension (OR = 1.12, 95% CI 1.08 to 1.16, *P* = 1.27 × 10^−11^) and high blood pressure diagnosed by doctor (OR = 1.14, 95% CI 1.09 to 1.18, *P* = 3.12 × 10^−11^) (Fig. 3, Additional file 2: Fig. S4). Analyses of treatment/medication conditions also showed that higher childhood BMI increased the risk of receiving blood pressure medication (Fig. 3, Additional file 2: Fig. S5). In contrast, we did not detect any association for acute myocardial infarction and varicose veins of lower extremities (*P* > 0.05 in all four MR methods; Additional file 1: Table S8). For the other traits, suggestive association signals were detected in at least one MR method, but the associations were no longer significant after multiple testing corrections.

#### Childhood BMI and endocrine, nutritional, or metabolic traits

We observed that a 1 SD increase in childhood BMI was associated with 36% higher odds of T2D (OR = 1.36, 95% CI 1.30 to 1.43, *P* = 1.57 × 10^−34^, Fig. 3, Additional file 2: Fig. S6). We also found evidence that higher childhood BMI caused increased risk the other 3 diabetes-related traits (Fig. 3, Additional file 2: Fig. S6). Higher childhood BMI also increased the risk of receiving Metformin, a drug for T2D treatment (Fig. 3, Additional file 2: Fig. S5). We observed adverse effects of childhood BMI on self-reported hypothyroidism (OR = 1.06, 95% CI 1.03 to 1.09, *P* = 8.77 × 10^−6^) and non-cancer thyroid problems (OR = 1.07, 95% CI 1.04 to 1.10, *P* = 7.78 × 10^−7^). For lipid traits, childhood BMI was negatively correlated with HDL cholesterol level (*β* = − 0.13, 95% CI − 0.19 to − 0.07, *P* = 1.33 × 10^−5^). The associations with triglycerides (*β* = 0.09, 95% CI − 0.04 to 0.14, *P* = 5.09 × 10^−4^) and total cholesterol (*β* = − 0.07, 95% CI − 0.12 to − 0.02, *P* = 8.11 × 10^−3^) were suggestive. No association between childhood BMI and LDL cholesterol level was detected (*P* > 0.05 in all four MR methods; Additional file 1: Table S8).

#### Childhood BMI and musculoskeletal system traits

As shown in Fig. 3 and Additional file 2: Fig. S7, we observed adverse effects of childhood BMI on self-reported osteoarthritis (OR = 1.07, 95% CI 1.05 to 1.10, *P* = 7.20 × 10^−8^), arthrosis (OR = 1.09, 95% CI 1.06 to 1.12, *P* = 8.80 × 10^−9^), and related traits. We also found evidence that childhood BMI was positively associated with adult heel bone mineral density (BMD) (details in Additional file 1: Table S2) (*β* = 0.20, 95% CI 0.15 to 0.24, *P* = 3.40 × 10^−20^).

#### Childhood BMI and other disease-related traits

As shown in Fig. 3 and Additional file 2: Fig. S8, we found evidence that higher childhood BMI caused an increased risk of cholelithiasis (OR = 1.26, 95% CI 1.18 to 1.35, *P* = 3.29 × 10^−5^), and the risk effect was supported by three MR methods (IVW, weighted median, and MR-Egger) after multiple testing corrections. Consistent with findings about general health, higher childhood BMI was also found to be associated with reduced health satisfaction (*β* = − 0.13, 95% CI − 0.18 to − 0.08, *P* = 7.44 × 10^−7^).

### Childhood BMI and adult lifestyle factors

As shown in Figs. 4 and 5, there is evidence that childhood BMI causally affects a total of 27 adult lifestyle factors, including 20 dietary habits, 4 smoking behaviors, usual walking pace (including three categories: slow pace (less than 3 miles per hour), steady average pace (3–4 miles per hour), and brisk pace (more than 4 miles per hour), details in Additional file 1: Table S2), pub/social club (a type of leisure/social activities, details in Additional file 1: Table S2), and alcohol intake frequency.

#### Childhood BMI and adult physical activities, smoking/drinking behaviors

As shown in Fig. 5 and Additional file 2: Fig. S9, for physical activities, we noticed that childhood BMI was negatively associated with usual walking pace (*β* = − 0.12, 95% CI − 0.15 to − 0.08, *P* = 3.24 × 10^−10^). For smoking behaviors, we observed positive associations between childhood BMI and adult smoking status. Higher childhood BMI was negatively associated with alcohol intake frequency (*β* = − 0.13, CI − 0.17 to − 0.09, *P* = 2.74 × 10^−11^).

#### Childhood BMI and adult dietary habits

We observed a positive association between childhood BMI and adult diet portion size (*β* = 0.26, 95% CI 0.18 to 0.34, *P* = 7.34 × 10^−11^, Fig. 5, Additional file 2: Fig. S10). In contrast, higher childhood BMI was associated with low calorie density food intake (Fig. 5, Additional file 2: Fig. S10). For example, childhood BMI was positively associated with the intake of high-fiber foods (e.g., fresh fruit intake, bran cereal, and wholemeal bread) and low fat/sugar food (e.g., skimmed milk, never/rarely using spread on bread, never eat sugar or food/drinks containing sugar). We also found negative associations between childhood BMI and the intake of meat (beef, lamb/mutton, and processed meat), full cream milk, and butter spread on bread.

### Childhood BMI and other traits

In the hematological test traits, we observed the positive association between childhood BMI and several traits associated with reticulocyte (e.g., reticulocyte percentage, *β* = 0.10, 95% CI 0.07 to 0.14, *P* = 9.15 × 10^−8^).

We did not observe significant association between childhood BMI and education qualification related traits (Additional file 1: Table S8). For socioeconomic status, we observed suggestive evidence that childhood BMI was negatively associated with average total household income before tax (*P* < 0.05 in IVW, weighted median and weighted mode methods but this association did not meet our significant criterion). We did not detect any association between childhood BMI and Townsend deprivation index at recruitment (a measure of material deprivation within a population which incorporates four variables: unemployment, non-car ownership, non-home ownership and household overcrowding, details in Additional file 1: Table S2) (*P* > 0.05 in all four MR methods; Additional file 1: Table S8).

### MR analyses in additional datasets without UK Biobank participants and PRS analysis for dietary habits in the UK Biobank data

We used several datasets (Additional file 1: Table S5) without UK Biobank participants to check whether the significant results could also be found in other studies. The results were consistent with our previous findings for disease-related traits (Additional file 1: Table S9, Additional file 2: Fig. S11). For example, childhood BMI was positively associated with disease count (*β* = 0.14, CI 0.06 to 0.22, *P* = 6.32 × 10^−4^). Higher childhood BMI increased the risk of CAD (OR = 1.10, CI 1.06 to 1.12, *P* = 1.20 × 10^−6^), hypertensive disease (OR = 1.21, CI 1.11 to 1.32, *P* = 1.33 × 10^−5^), T2D (OR = 1.18, CI 1.12 to 1.24, *P* = 8.85 × 10^−11^), and osteoarthritis (OR = 1.16, CI 1.06 to 1.26, *P* = 1.04 × 10^−3^).

We could not find available summary data in the European population for the other significant traits. Specifically, for dietary habits, we carried out MR analysis using adult BMI as exposure and 3 dietary habits from the Asian population as outcomes instead. Sixteen SNP instruments (Additional file 1: Table S10) were selected from the GWAS study by Wen et al. [58] in 86,757 Asians recruited from 21 studies. The outcome data were published by Matoba et al. [59], including up to 165,084 Japanese individuals collected by Biobank Japan (Additional file 1: Table S11). As shown in Additional file 1: Table S11, significant positive association between adult BMI and coffee intake was observed (*β* = 0.17, CI 0.11 to 0.24, *P* = 1.08 × 10^−7^). However, no association was found between adult BMI and meat/vegetable intake (*P* > 0.05).

We further carried out PRS analysis in the UK Biobank population. As shown in Additional file 1: Table S12, PRS for both childhood BMI and adult BMI is associated with higher portion sizes, more fruit intake and other low calorie density food intake, the direction of which was the same as the MR analysis.

### Multivariable MR analyses

#### The independent effects of childhood BMI after accounting for adult BMI

For the 60 outcomes with significant MR analysis results, we also carried out MR analyses for adult BMI. As it might be expected, although the effect sizes were different, at least suggestive associations (*P* < 0.05) were detected between adult BMI and these traits (Additional file 1: Table S13). The results were similar to the results of Millard et al. [12]. We performed multivariable MR analyses to assess the causal effects of childhood BMI which might be independent of adult BMI. As shown in Additional file 1: Table S14, after accounting for adult BMI, the effects of childhood BMI on adult traits were attenuated or no longer present. At the significant level of *P* < 0.05, we detected the associations between childhood BMI and 14 traits, including 12 dietary habits, heel BMD, and reticulocyte percentage. Of note, the detrimental effects of childhood BMI on disease-related traits (e.g., CAD, T2D, and arthrosis) were no longer present (*P* > 0.05).

#### Positive association between adult BMI and heel BMD was no longer present after accounting for childhood BMI

We also analyzed whether the effects of adult BMI are independent of childhood BMI. As shown in Additional file 1: Table S15, at the significant level of *P* < 0.05, the associations between adult BMI and 70% (42/60) of the traits remained after accounting for childhood BMI. Of note, while the positive association between childhood BMI and heel BMD was significant after accounting for adult BMI (*β* = 0.11, CI 0.02 to 0.20, *P* = 0.0211, Additional file 1: Table S14), the association between adult BMI and heel BMD was no longer exist after accounting for childhood BMI (*P* > 0.05, Additional file 1: Table S15).

### Reverse-direction MR analyses

The independent outcome number for the 60 traits was 33, setting the Bonferroni *P* value threshold for the main MR analysis at *P* < 1.52 × 10^−3^ (0.05/33). Similar to the forward MR analysis, confident results supported by both main MR method and weighted median MR method were considered as significant. As shown in Additional file 1: Table S16, we did not detect significant association for childhood BMI. Significant associations between 6 traits and adult BMI were detected (Additional file 1: Table S17 and Additional file 2: Fig. S12), including 3 diabetes traits, overall health rating (*β* = − 0.36, CI − 0.45 to − 0.27, *P* = 2.41 × 10^−14^), alcohol intake frequency (*β* = − 0.30, CI − 0.40 to − 0.21, *P* = 6.86 × 10^−10^), and usual walking pace (*β* = − 0.25, CI − 0.38 to − 0.13, *P* = 3.53 × 10^−5^). In addition, we observed suggestive positive association between portion size and adult BMI (*β* = 0.22, CI 0.06 to 0.37, *P* = 6.09 × 10^−3^).

## Discussion

In this study, with GWAS summary data from public resources, we carried out two-sample MR analyses to investigate the causal effects of childhood BMI on adult outcomes with genetic correlation. We identified potential causal effects of childhood obesity on 60 adult traits. Compared with previous studies of childhood BMI which only focused on a few traits [10, 11], here we provided a phenome-wide investigation of the causal associations between childhood BMI and adult outcomes.

### Childhood obesity is a risk factor for general health outcomes in adulthood

We observed that childhood obesity is a risk factor for general health outcomes in adulthood. Consistently, previous studies have demonstrated that high childhood BMI was associated with increased mortality and morbidity [2] in adulthood.

Specifically, we observed adverse effects of higher childhood BMI on CAD and T2D. This is consistent with the results of a previous MR study by Geng et al. [10]. We also replicated their finding about the negative association between childhood BMI and HDL cholesterol level, which is a well-known trait inversely related with CAD [60]. In addition, positive association between childhood BMI and high blood pressure was supported by different MR methods. Our analyses on treatment/medication conditions further showed that higher childhood BMI increased the risk of receiving CAD and T2D related medications, including blood pressure medication and metformin. Observational studies have also shown that higher childhood BMI is related to increased incidence of diabetes [61], CAD [3], and hypertension [62]. These data supported that childhood obesity might be a determinant of adult CAD/T2D risk.

Consistent with another MR study on childhood BMI [11], we detected positive association between childhood BMI and adult osteoarthritis, especially hip and knee pain. A previous observational study suggested that obesity from childhood had an accumulative effect on knee osteoarthritis development [63]. Similarly, a study by McFarlane et al. [64] on the 1958 British birth cohort observed a significant association with knee pain at the age of 45 years with high BMI from as early as age 11 years [64]. Moreover, another study [65] reported that the childhood overweight measures were significantly associated with adulthood knee mechanical joint pain among males. Therefore, it is possible that the effect of childhood obesity on the knee joint can persist into adulthood.

### The adverse effects of childhood BMI on disease-related traits were no longer present after accounting for adult BMI

Our multivariable MR analysis results showed that the positive associations between childhood BMI and increased risks of adult diseases (e.g., CAD, T2D, and arthrosis) were no longer present (*P* > 0.05) after accounting for adult BMI. Consistently, Richardson et al. [17] showed that the causal adverse effects of large body size in early life on CAD and T2D is depend on adult body size. A recent observational study [15] has shown that the association between childhood overweight and adult T2D only holds if the overweight continued until puberty or later ages. These findings suggest that there is a window of opportunity to mitigate the detrimental impact of childhood obesity. Indeed, a previous study [66] observed reversal of T2D and improvements in cardiovascular risk factors after surgical weight loss in adolescents. Therefore, ensuring that childhood obesity does not persist into later life might be useful for reducing the detrimental effects of childhood obesity on adult diseases. On the other hand, since 70% of obese adults were not obese in childhood or adolescence [67], targeting obesity reduction in adults is still very important to reduce the overall burden of obesity.

### The significant association between higher childhood BMI and low calorie density food intake in adulthood

For dietary habits, it was unexpected that higher childhood BMI was associated with low calorie density food intake. However, positive associations between childhood obesity and healthy diet habits have been reported in observational studies previously. For example, a healthy diet score was associated with increased odds of overweight/obesity in children from the UK [68]. Similarly, less frequent intake of energy-dense foods was associated with larger waist circumference in Swedish children [69]. It is possible that subjects suffering from childhood obesity may reduce their intake of unhealthy foods to lose weight.

The PRS analysis using UK biobank data also detected the association between higher childhood/adult BMI and low calorie density food. However, our MR analysis in the Asian population did not find any significant association between adult BMI and meat/vegetable intake. Therefore, it is likely that the association between BMI and low energy dense food is specific to the UK biobank population. Our current results might be affected by the fact that the enrolled individuals in the UK Biobank demonstrated a “healthy volunteer bias” [70], with lower rates of obesity and fewer self-reported health conditions than the general population.

### Positive association between adult BMI and heel BMD was no longer present after accounting for childhood BMI

We observed a positive association between childhood BMI and adult heel BMD. A previous MR study reported that adiposity is causally related to increased BMD at all sites except the skull in 5221 subjects from the Avon Longitudinal Study of Parents and Children [71]. In adults, MR analysis suggested that adiposity might be causally related to BMD at the femur [72]. Protective effect on osteoporosis of higher BMI in adults has also been reported previously [73]. We also observed a positive association between adult BMI and adult heel BMD. However, after accounting for childhood BMI, the positive association of adult BMI and heel BMD vanished, suggesting that this association depend on childhood BMI. It is widely accepted that most of the skeletal mass is acquired by the age of 20. Several studies have suggested that peak bone density is achieved by the end of adolescence [74, 75]. The risk of developing osteoporosis is influenced to a large extent by the levels of peak BMD. Our results implicated that the increasing effect on BMD of obesity might mainly work in childhood. Investigations taking peak BMD into consideration in adults are further needed to confirm our findings.

### The reverse-direction causal effects

In the reverse-direction MR analyses, we did not detect significant association between the 60 traits and childhood BMI. Meanwhile, 6 traits were detected to be causally associated with adult BMI. For example, we noticed that diabetes diagnosed by doctor was negatively associated with adult BMI. This might be as expected since lipolysis, proteolysis, and acute fluid loss during diabetes could cause weight loss [76]. Of note, we detected a negative causal effect of alcohol intake frequency on adult BMI. Consistently, Tolstrup et al. [77] reported that obesity was inversely associated with drinking frequency for a given level of total alcohol intake. A previous study [78] on alcohol-dependent individuals reported that subjects consuming the highest levels of alcohol had decreased fat mass. In addition, high alcohol consumption might impair nutrient absorption [79]. However, while frequently drinking moderate amounts of alcohol may protect individuals from weight gain, heavy drinking is more consistently related to weight gain [80]. In forward MR analysis, a causal negative effect of adult BMI on alcohol intake frequency was detected. These results highlight a bidirectional relation between obesity and alcohol intake. Further studies are needed to detail the mechanism link between obesity and alcohol consumption. We also detected a negative causal effect of usual walking pace on adult BMI. Forward MR analysis showed a causal negative effect of adult BMI on usual walking pace. Previous studies have reported that obese adults prefer to walk at a slower speed than their lean counterparts [81, 82]. As a most common type of physical activity in daily life, walking is the principal component of non-exercise activity thermogenesis [83]. Since higher levels of physical activity are consistently associated with weight loss maintenance [84], increasing usual walking speed may be an active and useful strategy for weight management.

### General limitations of the study

The limitations of the current study should be addressed. Firstly, because there are inevitably overlapping loci between childhood BMI and adult BMI, it is hard to identify which of these causal effects are due to early-life obesity, as opposed to late-life effects. However, childhood BMI GWASs conducted to date are notably smaller in sample size compared to adulthood GWASs; it is hard to obtain variants only associated with childhood BMI and not with overall BMI. When data for larger scale GWASs on childhood BMI are available, the power will be improved to identify more SNPs specifically associated with childhood BMI with smaller effects, and then the results of our analysis might be updated. Secondly, although our analyses supported that our results were not affected by pleiotropy, we cannot rule out the possibility of a shared genetic basis rather than a causal relationship. Thirdly, since we used GWAS summary data from the public database for our analyses, we cannot assess the effects of population stratification on our results. Summary data from multiple multi-ethnic populations might lead to biased association results since different ethnic populations have different LD structures and allele frequencies [85]. The summary data we used here were mainly derived from the European population. However, since we did not subset to the European-only results, there is a potential of bias from significant distinctions in disease outcomes between European and non-Europeans. UK Biobank is an unparalleled resource of extensive health information from 500,000 individuals [86]. Over 95% of our results are derived from the UK Biobank population. However, the UK Biobank data were reported to be skewed as wealthier and more educated [70]; this might affect the generalization of our results. Lastly, we did not take sex into account in both exposure and outcomes. Besides, clinical and public health decisions about potential interventions ideally require evidence about the effect size of the exposure on outcomes. However, this must be approached with care since Mendelian randomization estimate the effects on outcomes of a lifelong exposure to exposure risk SNPs, rather than an intervention at a specific time in life for a specific duration [22]. Therefore, the effect sizes from MR analyses in our study should not be considered equivalent to those from an RCT of a short-term intervention [87].

## Conclusions

In summary, using public GWAS datasets, we carried out 2-sample MR analyses to investigate the causal effects of childhood BMI on adult outcomes. We identified potential causal effects of childhood obesity on 60 adult traits. Our results suggested that the adverse effect of obesity might start early from childhood, but the positive association between childhood BMI and diseases-related traits in adulthood can be attributed to individuals remaining obese in later life.

## Supplementary Information

**Additional file 1.** This file provides the details of Supplementary tables S1-S17.

**Additional file 2.** This file provides the details of Supplementary figures S1-S12.

## Data Availability

The childhood BMI dataset was downloaded from the Early Growth Genetics Consortium (http://egg-consortium.org/childhood-bmi.html, “EGG_BMI_HapMap_DISCOVERY.txt.gz”). The 903 adult outcome datasets were from the Genome-wide Complex Trait Analysis (GCTA) website (https://cnsgenomics.com/software/gcta/#DataResource), the Gene ATLAS database (http://geneatlas.roslin.ed.ac.uk/), and the LDhub GWAShare Center (http://ldsc.broadinstitute.org/). Details for each dataset can be obtained from Additional file 1: Table S2. The replication datasets (details in Additional file 1: Table S5) were from the GERA cohort [49] and the studies performed by Nikpay et al. [50] and Scott et al. [51]. The UK Biobank data were obtained under the application number 46387.

## Abbreviations

BMI: Body mass index
MR: Mendelian randomization
CAD: Coronary artery disease
T2D: Type 2 diabetes
LD: Linkage disequilibrium
IVW: Inverse variance weighted
INSIDE: Instrument strength independent of the direct effects
NOME: No measurement error in the SNP exposure effects
SIMEX: Simulation extrapolation
MR-PRESSO: MR pleiotropy residual sum and outlier
PRS: Polygenic risk score
