# Supplementary material for: Phenome-wide investigation of the causal associations between childhood BMI and adult trait outcomes: a two-sample Mendelian randomization study
Source: Genome Med. 2021 Mar 26;13:48. doi: 10.1186/s13073-021-00865-3 (PMC8004431; doi:10.1186/s13073-021-00865-3)
Supplement: Supplementary file 2 — Additional file 2. This file provides the details of Supplementary figures S1-S12. [file 13073_2021_865_MOESM2_ESM.pdf]

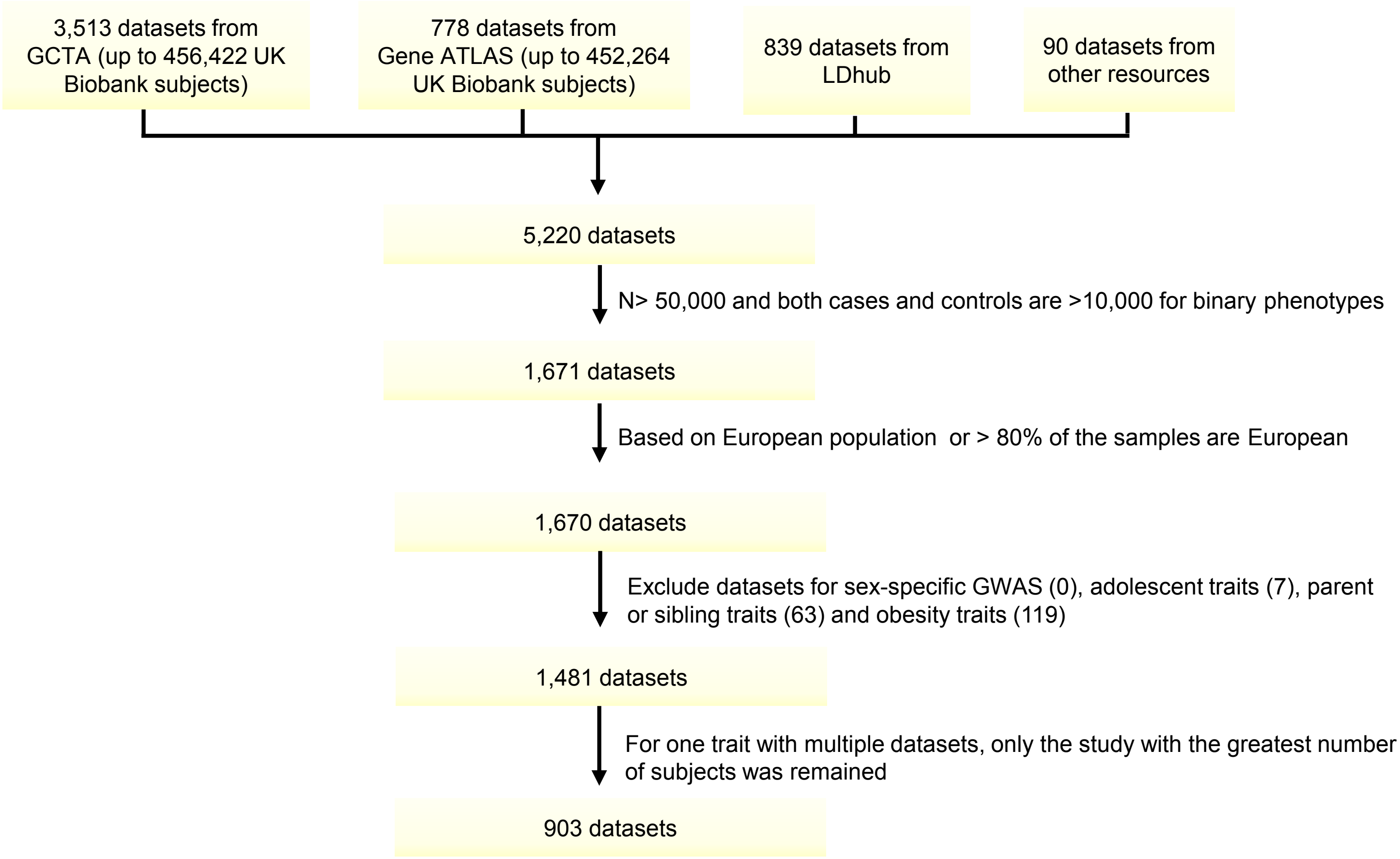

Fig. S1 The data filtering process.

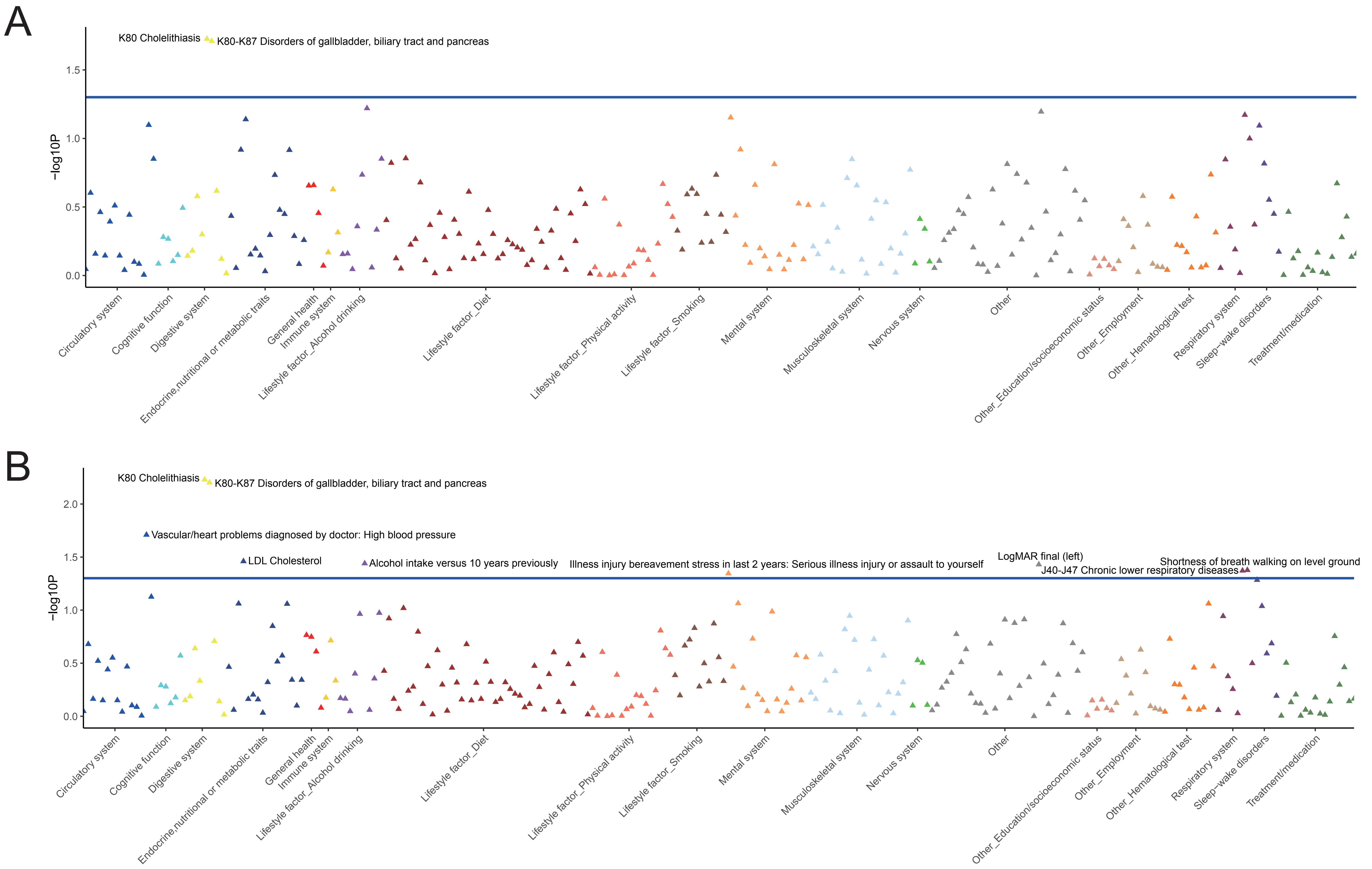

Fig. S2 Manhattan plot showing the pleiotropy assessment results for MR-Egger intercept test (A) and Q-Q' test (B). For each plot, the blue horizontal line represents the threshold for significance (P< 0.05).

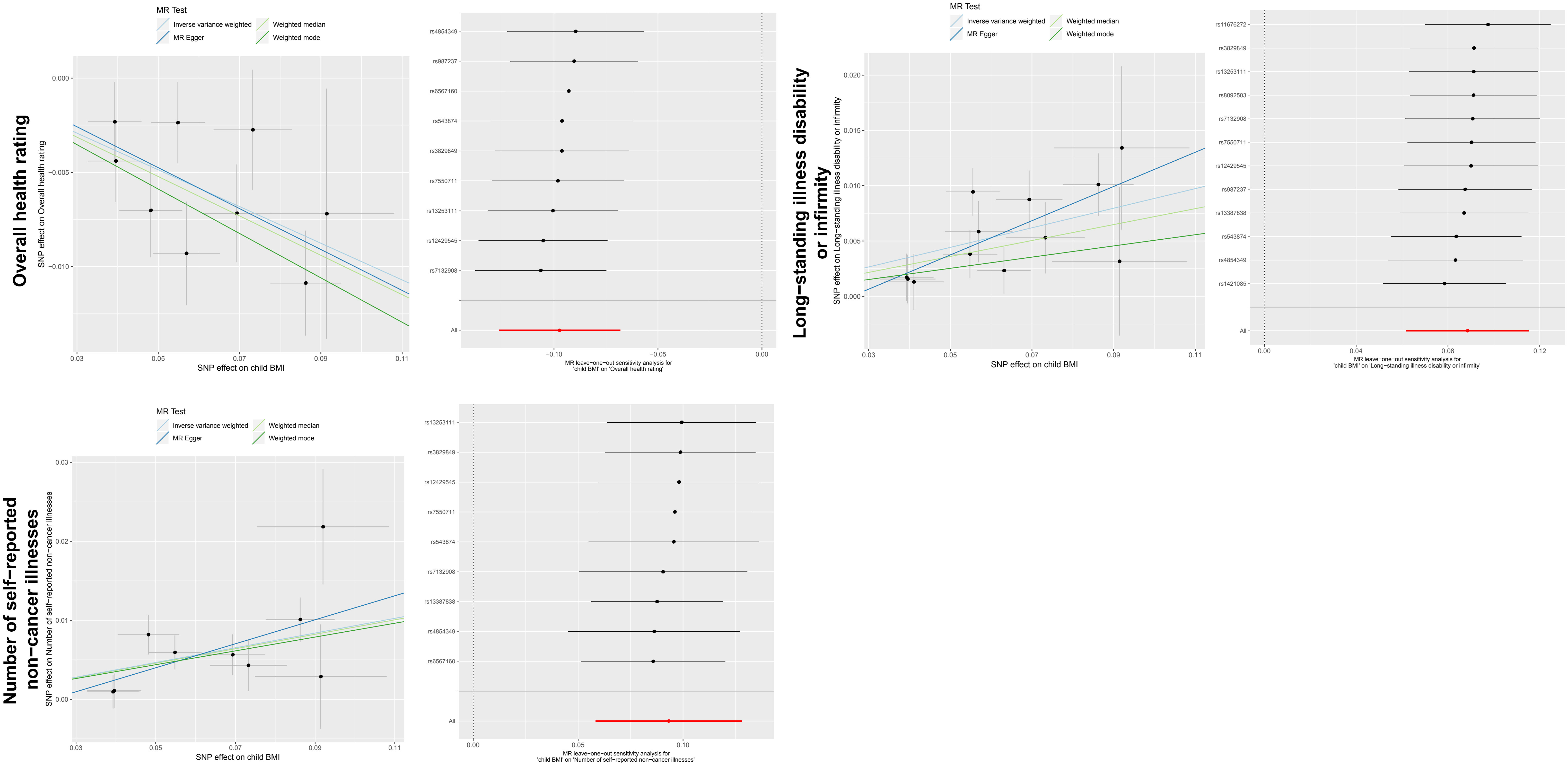

Fig. S3 Scatter plot and leave one out analysis plot for overall health rating, long−standing illness disability or infirmity, and number of self−reported non−cancer illnesses.

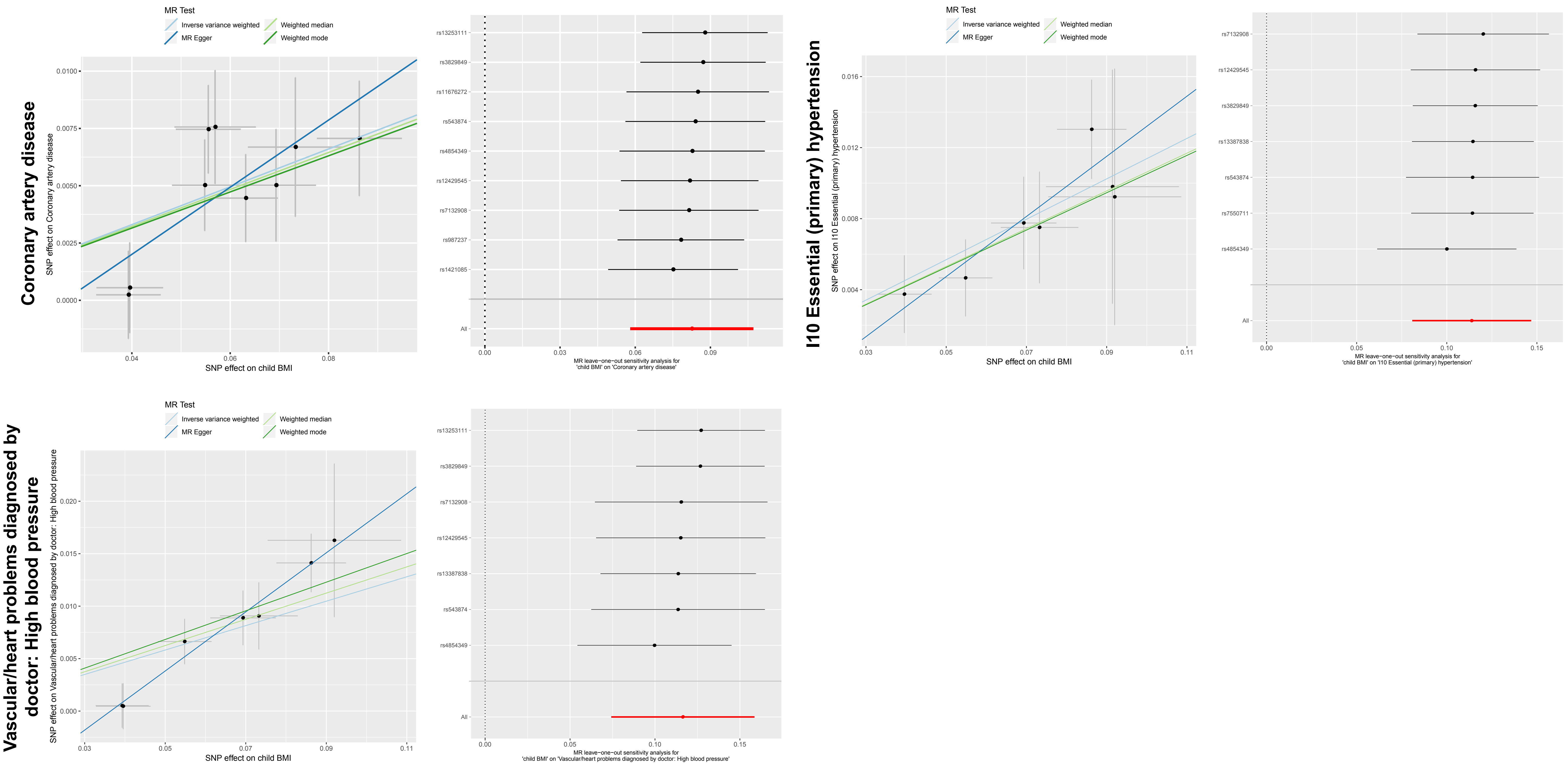

Fig. S4 Scatter plot and leave one out analysis plot for circulatory system traits.

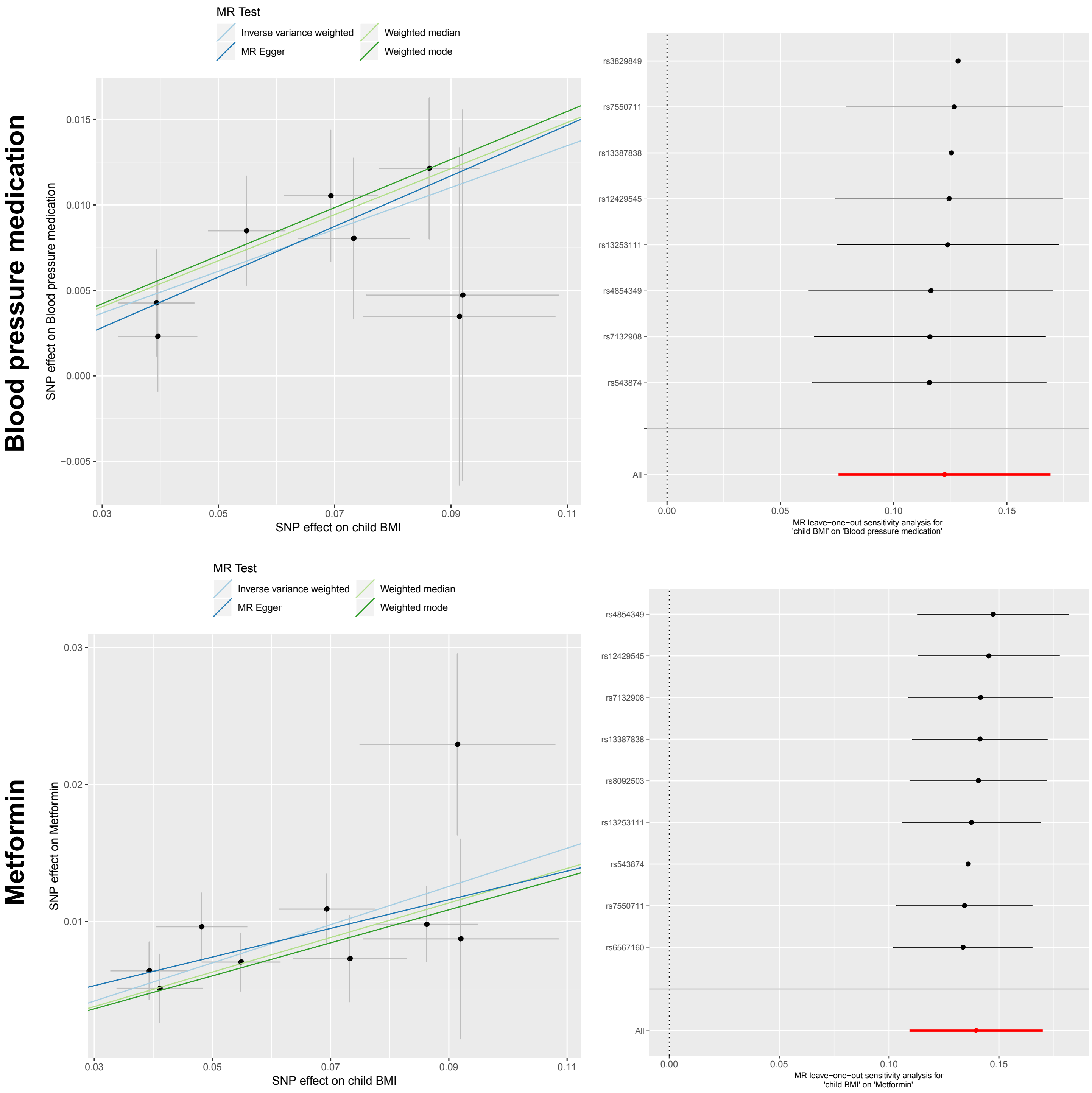

Fig. S5 Scatter plot and leave one out analysis plot for traits of received treatment/medication.

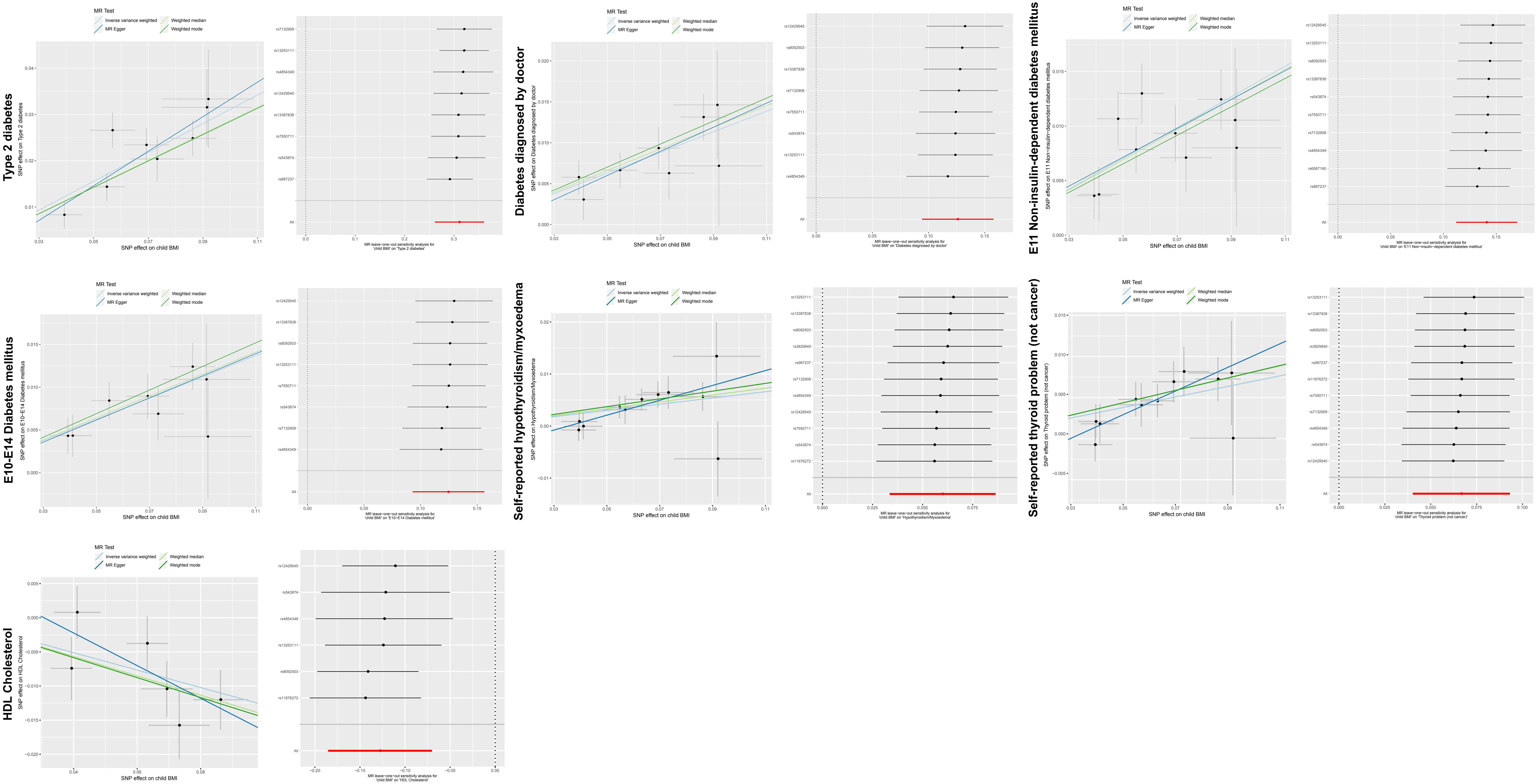

Fig. S6 Scatter plot and leave one out analysis plot for endocrine, nutritional or metabolic traits.

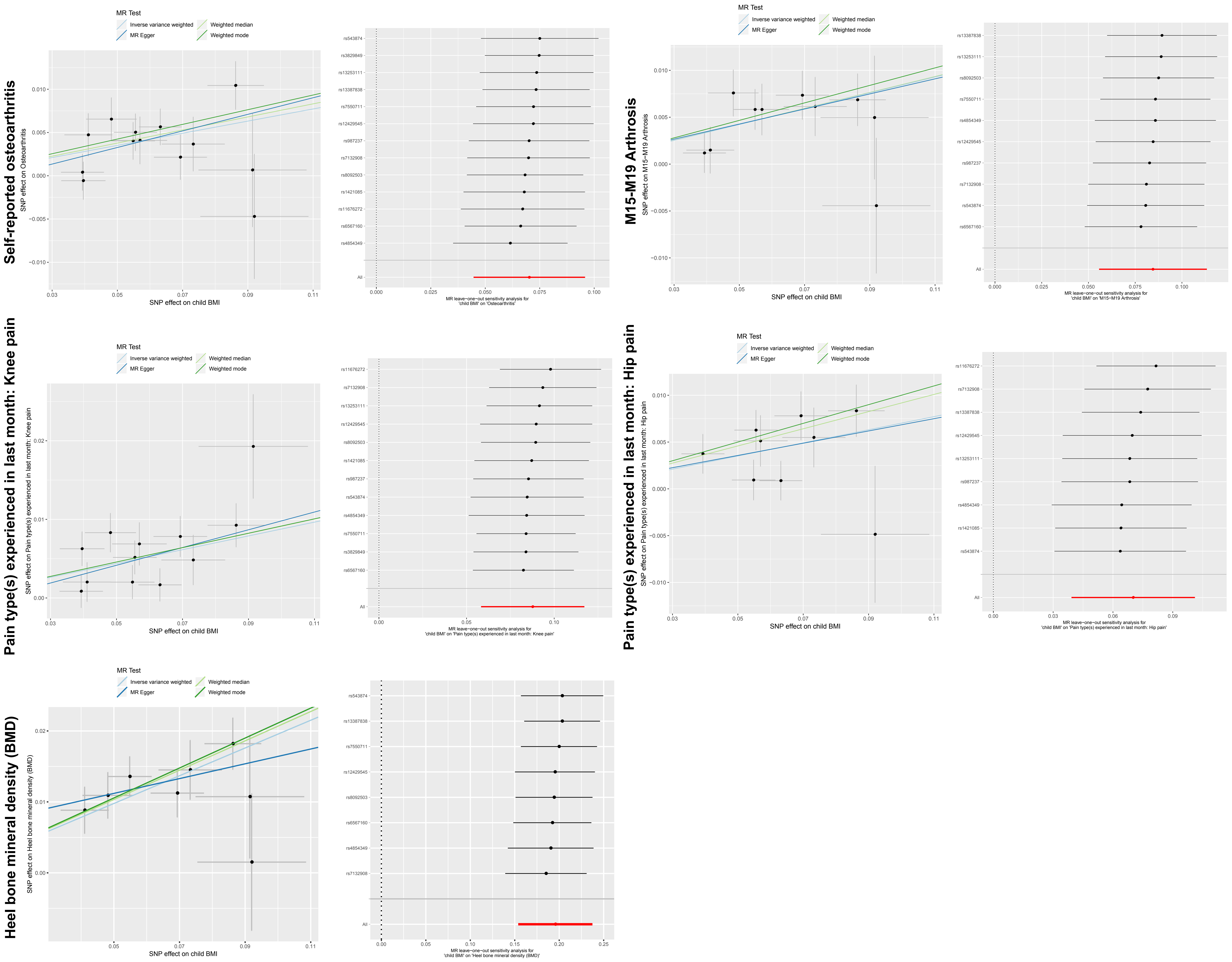

Fig. S7 Scatter plot and leave one out analysis plot for musculoskeletal system traits.

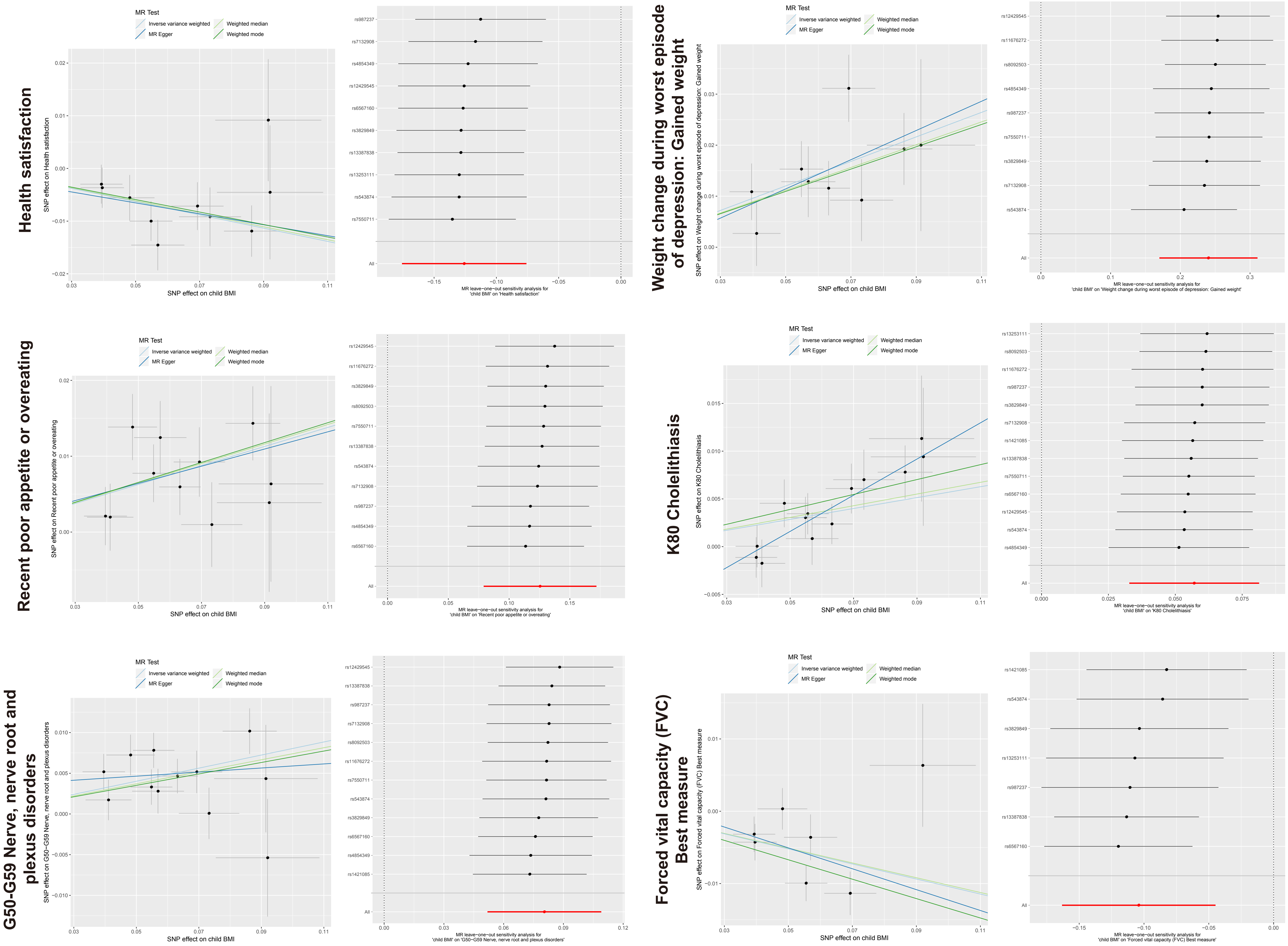

Fig. S8 Scatter plot and leave one out analysis plot for the rest disease-related traits.

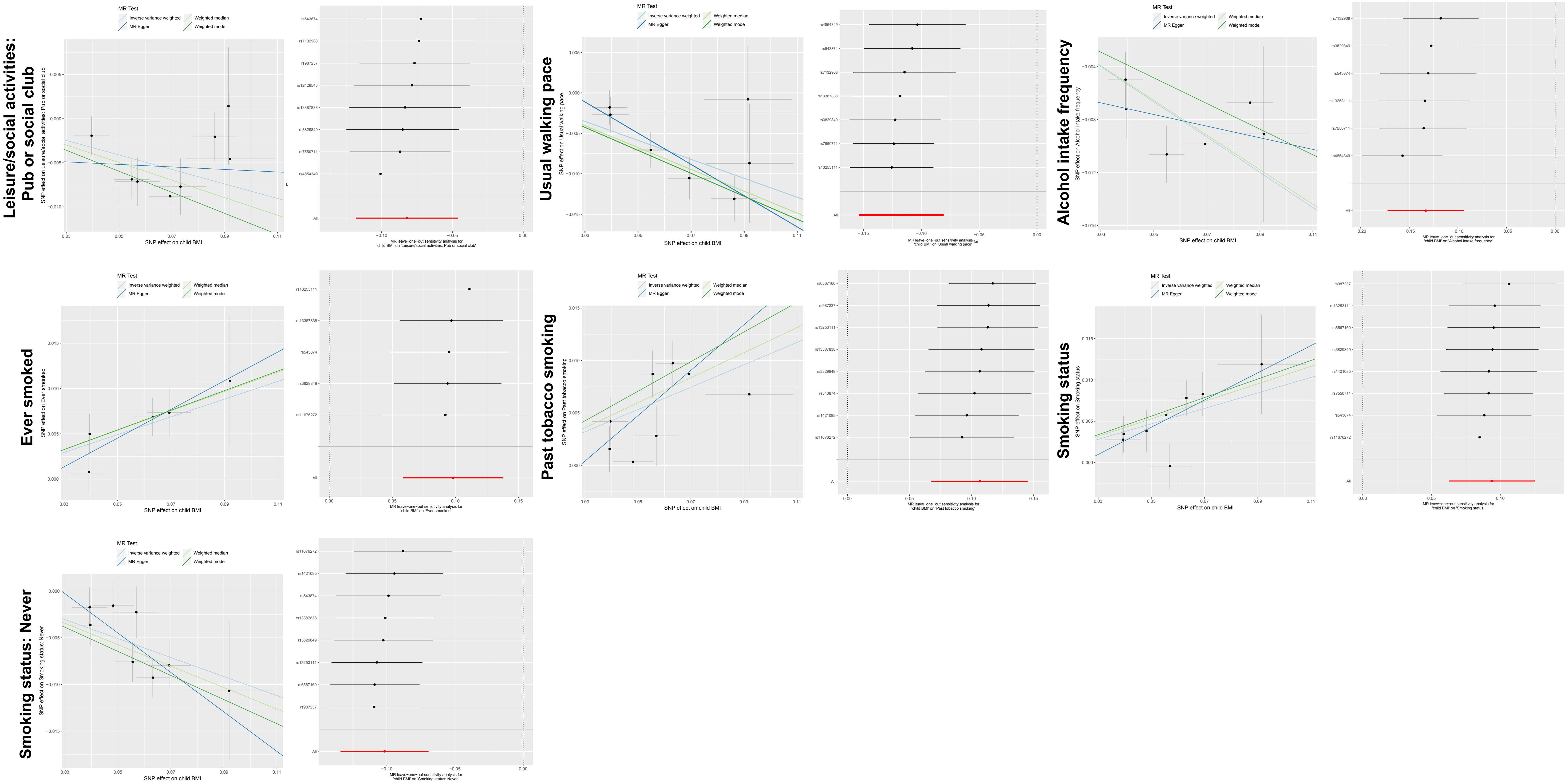

Fig. S9 Scatter plot and leave one out analysis plot for physical activity, smoking behaviors, and alcohol intake frequency.

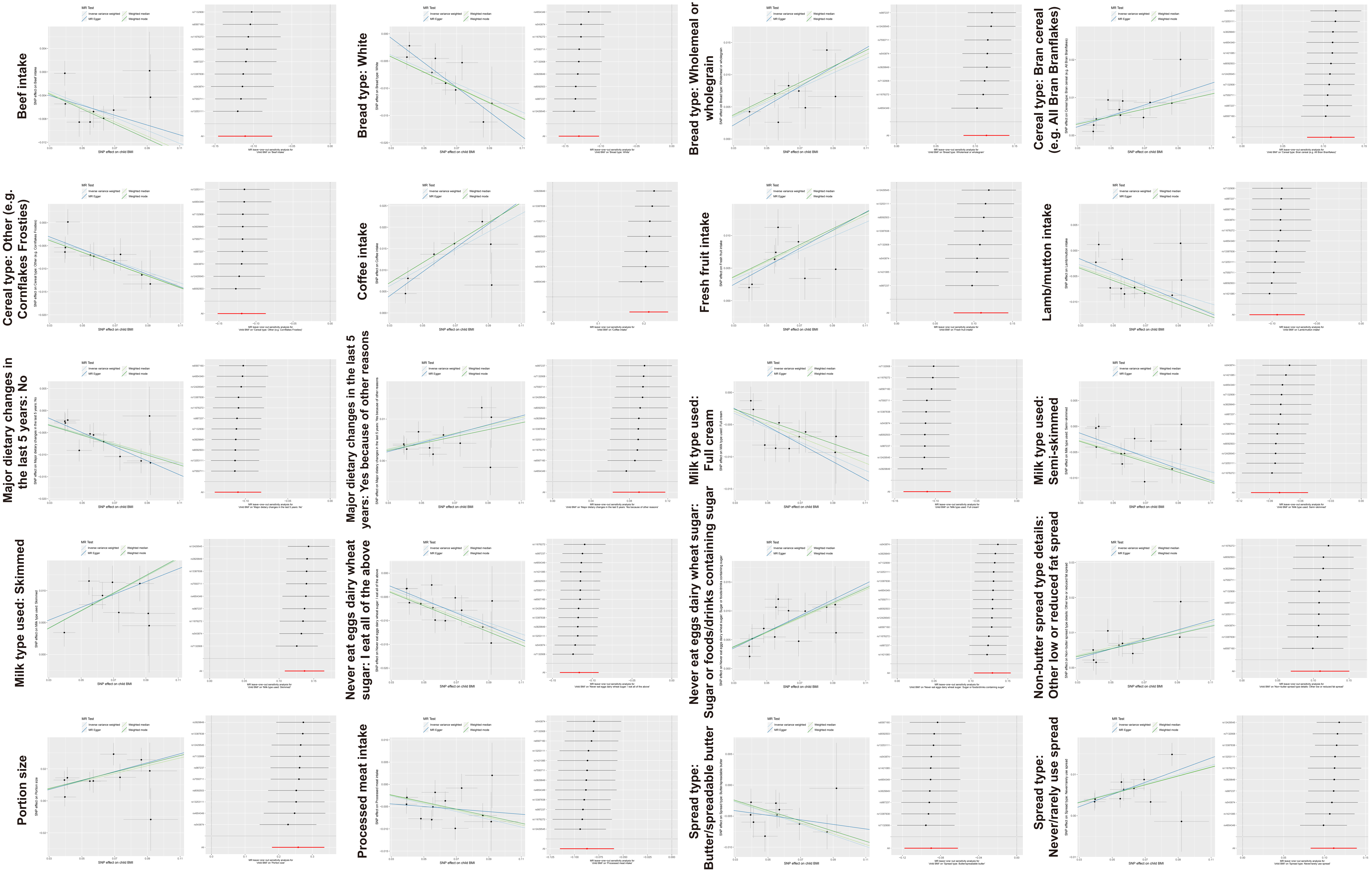

Fig. S10 Scatter plot and leave one out analysis plot for dietary habits.

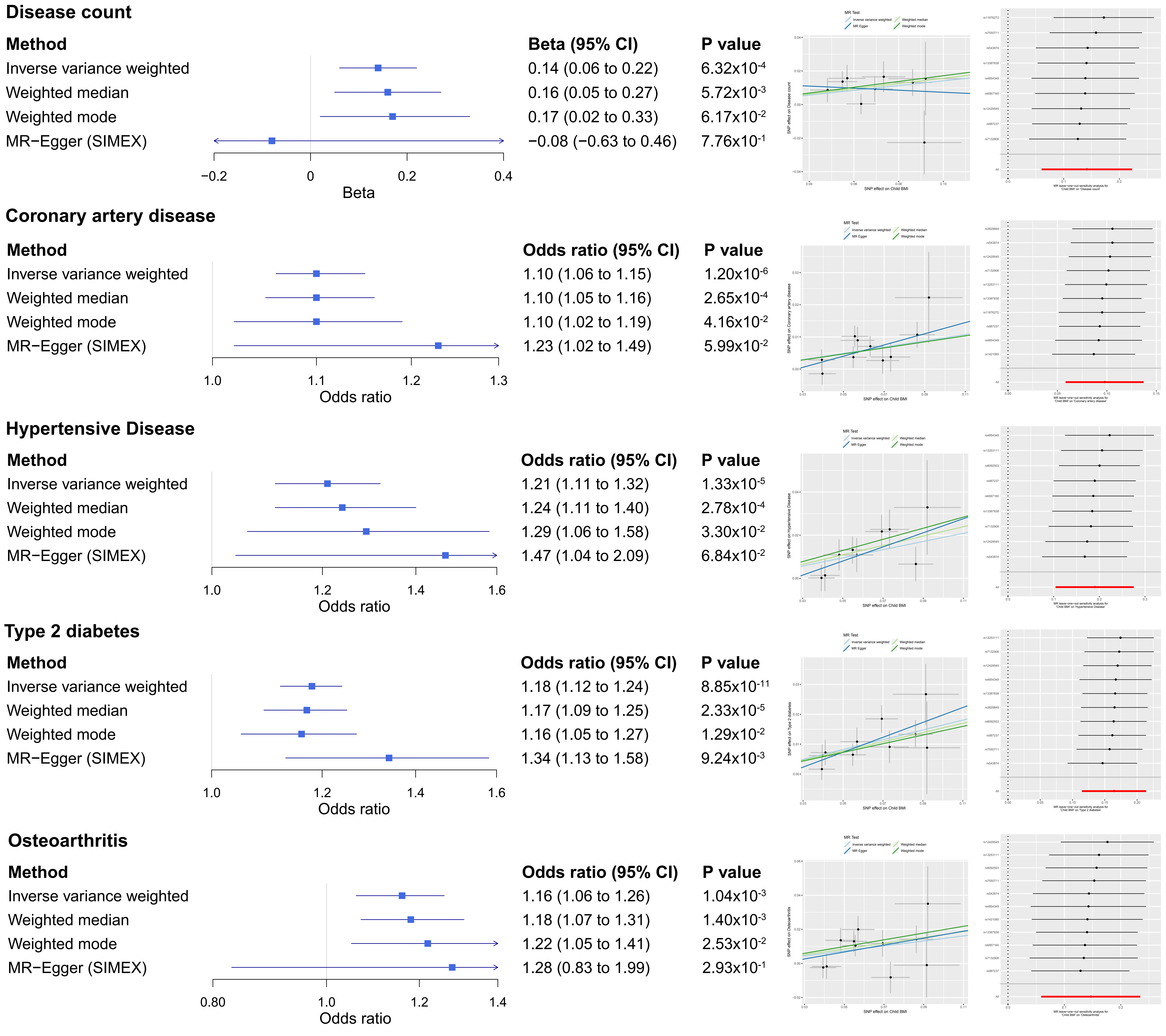

Fig. S11 Summary Mendelian randomization (MR) estimates derived from the inverse-variance weighted, MR-Egger, weighted median and weighted mode -based methods for additional datasets. Childhood BMI was used as exposure and significant associations were detected for these traits. Scatter plot and leave one out analysis plot for each trait are also shown.

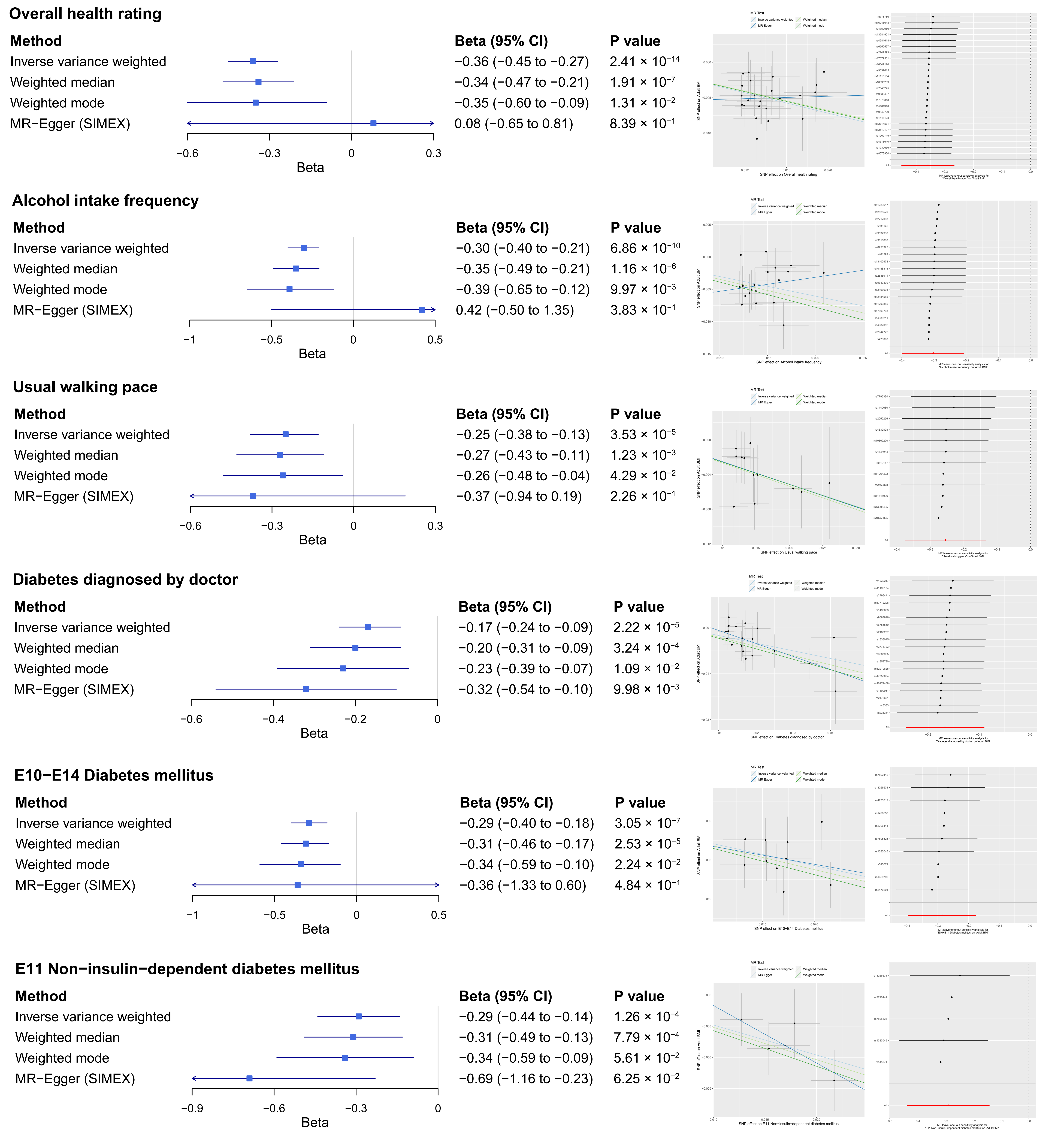

Fig. S12 Summary Mendelian randomization (MR) estimates derived from the main inverse-variance weighted, MR-Egger, weighted median and weighted mode-based methods for reverse analyses using adulthood BMI as outcome. Scatter plot and leave one out analysis plot for each trait are also shown.
